# Development, validation, and reliability of the Irrational Beliefs Scale for Sports Officials (IBSSO)

**DOI:** 10.3389/fpsyg.2025.1571447

**Published:** 2025-04-10

**Authors:** Stuart C. Carrington, Martin James Turner, Jamie S. North, Abbe Brady

**Affiliations:** ^1^Faculty of Sport, Technology and Health Science, St Mary’s University, Twickenham, London, United Kingdom; ^2^Manchester Metropolitan University, Manchester, United Kingdom

**Keywords:** confirmatory factor analysis, referees, rational-emotive behavior therapy, exploratory factor analysis, officials

## Abstract

Application of Rational-Emotive Behavior Therapy (REBT) within performance environments is increasing, however measures that assess irrational beliefs in specific populations are encouraged. A population that may benefit from REBT is sports officials. This paper reports the development, validation and reliability of the Irrational Beliefs Scale for Sports Officials (IBSSO). Item development was drawn from original items of the Irrational Performance Beliefs Inventory (iPBI), then refined over three stages using an expert panel, novice panel and industry panel. Officials (*N =* 402; 349 male, 50 female, 3 undisclosed) from 11 sports (*M* years’ experience = 13.02; *SD =* 10.24) completed the inventory, with exploratory factor analysis suggesting a 3, 4, and 5-factor model from 22 remaining items. A new sample of 154 officials (140 male, 12 female, 2 undisclosed) representing 9 sports (*M* years’ experience = 14.61, *SD* = 11.96) completed the IBSSO, along with 6 other related measures (e.g., Automatic Thoughts Questionnaire, Affective Reactivity Index) to assess criterion validity. A four-factor model showed acceptable fit, with self-depreciation, peer rejection demands, emotional control demands, and approval identified as subscales, as well as a three-factor model. The IBSSO was positively correlated with the additional measures and negatively correlated with age, demonstrating concurrent validity. To assess convergent validity, 94 new officials (83 male, 10 female, 1 undisclosed; *M*_age_*=* 36.74 years, *SD* = 15.03) completed the IBSSO and iPBI simultaneously. The IBSSO was positively correlated with the iPBI, indicating convergent validity. Furthermore, 29 officials (25 male, 4 female, *M* years’ experience = 14.57, *SD* = 12.44) completed the IBSSO over three-time points, with a repeated-measures MANCOVA and Intra-Class Coefficients confirming test–retest reliability. The 16-item four-factor model was accepted based on statistical and theoretical fit. The paper presents a measure of irrational beliefs in sports officials, with investigation into the effectiveness of REBT with this population recommended.

## Introduction

Adverse events are an inevitable part of life. Although the impact of adverse events on undesirable emotions (e.g., depression and anxiety) is stable between-persons (i.e., unfortunate events promote undesirable emotions), the relationship within-persons is bidirectional (e.g., depressed individuals are influenced by their environment but also actively shape it; Maciejewski et al., 2021). The bidirectional relationship between the individual and their environment on emotional outcomes is consistent with the fundamental assumption of Rational Emotive Behavior Therapy (REBT; Ellis, 1957), namely that it is not an adverse situation in isolation that promotes maladaptive behaviors and emotions, but rather the individual’s beliefs about that situation. As the elimination of negative events is impractical, unavoidable, and perhaps undesirable (see Seery, 2011), increased discussion and application of strategies to modify undesirable emotional responses to adverse events is worthwhile.

**Figure 1:**
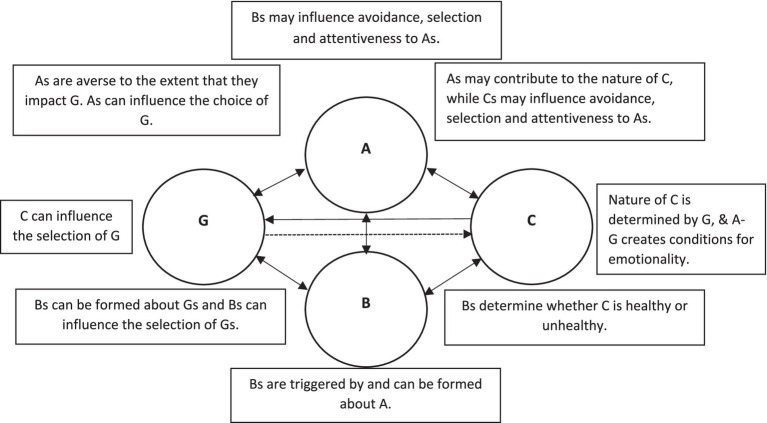
The interdependent GABC framework. Dotted line reflects the more distal relationships between G and C due to mediating effect of A that promotes negative emotion, not the G in isolation (adapted from Turner, 2022). G, goal; A, adverse event; B, beliefs; C, consequences (emotional and behavioral).

REBT is situated within the psychotherapeutic approach of cognitive behavioral therapy and is focussed on the role played by cognitions in emotional (and behavioral) responses. Consequently, it rejects traditional “Adversity-Consequence” (A–C) models to explain responses to negative situation and adopts an interactive “Goals-Adversity-Beliefs-Consequence” (GABC) model (see Figure 1). In this model, adverse events (A) to one’s goals (G) are met with unhealthy or healthy negative emotions (C), depending on one’s beliefs (B).

According to REBT the beliefs that create unhelpful emotions (e.g., anxiety) are labeled irrational as the subsequent behaviors they promote (e.g., avoidance or withdrawal) do not assist an individual in reaching their goal (Turner, 2022). Dryden and Branch (2008) identified four irrational beliefs: one primary belief (demands) and three secondary beliefs (awfulizing, self-deprecation and low-frustration tolerance). In contrast, beliefs that promote adaptive emotions and behaviors (e.g., concern and dealing with the perceived threat) are identified as rational as they assist the individual in goal achievement (Ellis and Dryden, 1997). Rational beliefs are identified as preferences (primary belief), anti-awfulizing, self-acceptance and high-frustration tolerance (secondary beliefs; Dryden, 2014; Dryden and Branch, 2008).

It is the promotion of adaptive emotions that has seen REBT successfully applied in a variety of contexts including education (e.g., Caruso et al., 2017), the military (e.g., Jarrett, 2013), clinical treatment (e.g., Hyland et al., 2013), and sport and exercise (e.g., Turner and Bennett, 2018). For instance, REBT has been credited with improving performance, increasing resilience, decreasing anxiety, and enhancing physical activity levels in elite and non-elite participants (Deen et al., 2017; O’Connor, 2018; Turner et al., 2020; Wood et al., 2017). A recommendation drawn from the successful application of REBT in sporting environments is for researchers to investigate and understand the benefits of REBT on other agents and stakeholders in sport and exercise (Jordana et al., 2020) such as sports officials.

Sports officials (e.g., referees, umpires and judges) have recently been the focus of increased attention from academic researchers (Hancock et al., 2021). However, research on sports officials has typically focussed on physical performance (e.g., Castillo, 2016, 2017; Weston et al., 2011), well-being (see Webb et al., 2021) and the use of video-training to develop decision-making skills (e.g., Samuel et al., 2019; Spitz et al., 2018). While video-training interventions have been shown to improve perceptual-cognitive skills (e.g., Helsen and Bultynck, 2004), such a reductionist approach fails to acknowledge the role of individual differences such as experience, social support and emotional regulation that mediates performance. For instance, Page and Page (2010) reported that the experience of sports officials mediates the impact of contextual constraints (e.g., crowd noise) on performance, while Praschinger et al. (2011) concluded that subjective “thresholds” for game management strategies (defined as the shift from accurate to adequate decisions; Raab et al., 2021) influenced decision-making following verbal abuse from players. Such differences mediate the impact of social constraints, such as crowd influence and player behavior, on officiating performance because of a desire to receive praise or avoid criticism (see Dohmen and Sauermann, 2016). Although research involving REBT and officials is in its infancy, four REBT sessions improved the performance of one rugby union official and decreased anxiety in two rugby union referees, with this improvement maintained over a 12-week period (Maxwell-Keys et al., 2022).

Minimizing unhealthy negative emotions, such as anxiety, may benefit performance by mediating situational appraisals. Irrational beliefs have been associated with increased threat appraisals (e.g., evaluation of future loss or harm), with rational beliefs associated with challenge appraisals (e.g., perceived future gain; Lazarus, 1991) due to an outcome being perceived as incongruent to goal achievement (Chadha et al., 2019; Lazarus, 1999). Promoting challenge appraisals may be especially helpful for sports officials, given the contextual constraints they operate within. Basketball officials, for instance, reported varying level of stress, challenge and threat appraisals depending on the time and score-line of a game (Ritchie et al., 2017). Although these situations were assessed using hypothetical scenarios, limiting the external validity of the findings, the identification of situations that cause officials to experience varying emotional outcomes is consistent with previous research. For example, Neil et al. (2013) demonstrated that adverse events promote maladaptive emotional and behavioral consequences in soccer referees (e.g., avoidance of appropriate disciplinary action for fear of potential conflict or criticism). Consequently, attempts to minimize maladaptive responses to negative events, such as “avoidance” (when an official takes, or does not take, action to achieve a desirable reaction from other agents; see Nevill et al., 2017) and self-depreciation (Mansell, 2021), are justified in pursuit of improved performance of sport officials. The focus of REBT on producing goal-congruent emotions and behaviors that mediate officiating performance (Bruch and Feinberg, 2017; Kostrna and Tenenbaum, 2022) alongside its short-term effectiveness, that is valued within officiating environments (Bowman and Turner, 2022; MacMahon et al., 2015), underlines the potential benefit of application in sports officials.

Irrespective of the population studied, however, there are important limitations of many traditional measures of irrational beliefs. Original measures of irrational beliefs, such as the Rational Behavior Inventory (Shorkey and Whiteman, 1977), contained items that assessed emotional and behavioral outcomes as well as beliefs (Ramanaiah et al., 1987). For instance, Robb and Warren (1990) demonstrated that the item, “I often get excited or upset when things go wrong,” assessed both the emotional outcome and its frequency of occurring following an adverse event. This is inconsistent with REBT theory, which states beliefs are the only cognitions that need to be assessed (Dryden and Branch, 2008). Such conceptual problems prompted Terjesen et al. (2009) to review psychometric measures of irrational beliefs and consequently recommend the need to develop a novel measure as, while some inventories reported good reliability (e.g., the Shortened General Attitude Belief Scale; SGABS, Lindner et al., 1999), all of the measures failed to provide a correlation with other measures of irrationality or cognitive disturbance, which would be expected, along with a lack of reporting around discriminant validity. Consequently, Terjesen et al. (2009) made the following recommendations regarding the development of an irrational beliefs inventory:

- items should only reflect the assessment of beliefs and not emotional or behavioral consequences
- items not only measure agreement/disagreement with irrational beliefs but also agreement/disagreement with rational beliefs
- items must contain roughly the same amount of the core irrational beliefs unless one is more clinically useful than others
- single item responses are avoided as they fail to account for the belief strength
- that the construct validity of the measure is not only reported but established *a priori* by having experts in REBT agree that items support the theoretical aims
- internal reliability should be measured and reported with a minimum Cronbach’s α of 0.70 met (Nunnally, 1972)

The development of the Irrational Performance Beliefs Inventory (iPBI; Turner et al., 2018a) aimed to address the recommendations made by Terjesen et al. (2009). The inventory was developed after consultation with an expert panel to generate, eliminate and/or amend any items that were not consistent with REBT theory. Specifically, items were sent to the expert panel in an Excel spreadsheet with drop down menus adjacent to each item. Expert panel members were asked to select which irrational belief and which subscale they believed the item measured. Additionally, there was an opportunity to add notes for each item, should the expert panel feel the item was ambiguous in any sense (theoretically, wording or otherwise). Following this step, a novice panel consisting of psychology graduates with little or no knowledge of REBT theory assessed the clarity, wording, and length of the items. Items measured agreement and disagreement of the four irrational beliefs (demands, low-frustration tolerance, self-depreciation and awfulizing) and account for belief strength by requesting responses on a Likert scale. The scale also reported internal consistency coefficients of between 0.90 and 0.96, exceeding the criteria for excellent test score reliability (Nunnally and Bernstein, 1994; Turner et al., 2018a). Importantly, the iPBI is a context specific measure. In this case, the chosen context is performance, which is relevant as the application of REBT has been shown to be effective in sporting and business environments (e.g., Turner and Barker, 2015; Turner and Bennett, 2018).

While the general context of performance featured within the iPBI is a benefit of the measure, a further, and important, recommendation for inventory development made by Terjesen et al. (2009) is that measures test specific populations. This endorsement is supported by Ziegler and Horstmann (2015), who advised that the construction of measures reflect theoretical advances that, while behaviors are influenced by individual personality, it is that individual’s *perception* of a specific situation that needs to be assessed (see Fleeson and Noftle, 2009; Funder, 2006). Therefore, adapting the iPBI for application involving target populations has three benefits. First, it would meet contemporary recommendations for bespoke measurement design, resulting in a higher signal-to-noise ratio and allow for greater awareness of the prevalence of irrational beliefs, a neglected area of research (Pargent et al., 2019; Turner, 2014). Second, it would provide insight into how contextually relevant situations are perceived by those directly involved, assisting populations who may benefit from interventions involving REBT (Jordana et al., 2020; Turner et al., 2018a; Ziegler and Horstmann, 2015). Finally, within the context of sports officials, the development of a measure that assesses how sports officials perceive relevant situations and their likely behavioral and emotional outcomes would assist in the desired holistic development of this population (e.g., Raab et al., 2021; Webb, 2017).

The aim of this study was to develop a version of the iPBI for specific and bespoke application with sport officials to provide REBT practitioners with a practical and contextually relevant tool for assessing core irrational beliefs with this population. A secondary aim was to establish the criterion and convergent validity of the new measure. As age and experience has been shown to reduce irrational beliefs (Ndika et al., 2012; Turner et al., 2019), it was hypothesized that younger and less experienced officials would report higher levels of irrational beliefs than their older and more experienced counterparts. A final aim was to assess test–retest reliability of the new measure.

### Preparatory work—item development

To develop and validate the measure, a three-stage process was undertaken and recorded using a decision-making log to enhance reporting accuracy. The first stage was item generation, the accuracy of which is essential to the development of a good measure (see Turner et al., 2018a). One hundred and thirty-three items from the original iPBI were reviewed and, if necessary, amended for consideration. Items were amended to enhance their relevance to the target population, as recommended by Terjesen et al. (2009), however it was necessary to ensure that items were not too specific. Avoidance of specificity is consistent with REBT theory (see Dryden and Branch, 2008; Ellis, 2005) which emphasizes the need to prevent a conditional “should/demand” to meet a future condition, such as “if I want to perform well as an official, it would be terrible to get important decisions wrong.” “To perform well” is a future condition, which is met, in part, with decision-making accuracy, whereas the scale’s purpose is to measure irrational beliefs. Therefore, an item relating to decision-making, identified as integral to the performance of sports officials (see Bruch and Feinberg, 2017; Samuel et al., 2020), was presented as “it’s terrible to fail by getting important decisions wrong,” measuring awfulizing, a secondary irrational belief. Such amendments increased relevance to sports officials as did the inclusion of two new subscales, control (14 items; 7 regarding self-control and 7 regarding control of others) and responsibility (7 items), reported as valued qualities in sports officials (see Carrington et al., 2022; Morris and O’Connor, 2017; Neil et al., 2013). Two of the authors, one of whom holds the Primary Certificate in REBT and one of whom holds the Advanced Practicum in REBT, reviewed each item. Following the same process that led to the development of the iPBI (Turner et al., 2018a), items were removed if they were judged to measure the behavioral or emotional response, rather than the belief, or if the primary belief and subscale measured could not be agreed upon. Consistent with the view of DeVellis (2012) that conservatism is the optimum approach for scale development, a policy adopted throughout the process, items that appeared repetitive or could not be amended to be more relevant for sports officials were retained, resulting in 90 items progressing to the second stage of development.

Following item development, the scale underwent review from three panels; expert, novice and a panel of experts from the target population (officials’ panel). This approach assists theoretical consistency, content validity and the possibility of refinement (Grant and Davis, 1997; Turner et al., 2018a). Experts (*n* = 3), each qualified REBT practitioners, were asked to assign an irrational belief (e.g., demand) and subscale (e.g., rejection) to each item. Four items were removed (“I cannot stand being snubbed by people I trust”; “I need to avoid failing in making important decisions”; “I need to be in control of others”; “I am no good if I do not succeed in controlling others”) following discrepancy across the expert panel regarding the irrational belief being measured. The remaining 86 items were subjected to review by a novice panel consisting of post-graduate psychology students (*n* = 4), with no training or grounding in REBT theory, to assess item clarity (see DeVellis, 2012). The panel reported that many items appeared to be repeated and that assessment of the “control” subscale was unclear. Following discussion with co-authors, similar items were retained to allow for statistical analysis and some items were refined to provide greater clarity (e.g., “if I am not in control then it means I am useless” became “if I am not in control of my emotions then it means I am useless”). An industry panel of football officials (*n* = 3, male = 2, female = 1, representing Level 1a, Level 3 and Level 5), were asked to review the clarity, appropriateness and relevance of the language used in each item to enhance social validity, defined as the social significance of goals and acceptability of potential intervention procedures (Common and Lane, 2017), within the target demographic. Eight items were reported as unclear. From these eight items, one item (“I would be useless if others threatened my status among my colleagues”) was removed due to incoherence reported by the officials’ panel. The other 7 items were amended where possible (e.g., the clarity of “it’s awful if others do not approve of me” was questioned as the opinion of some was considered more important than others, and so became “it’s awful if others that are important to me do not approve of me”), leaving 85 items, including 22 unchanged items from the iPBI.

The final stage involved repeating the panel process with one member of each panel to evaluate the clarity and validity of the remaining items. Prior to this stage, items assessing demands were amended to include the preference (e.g., “I need others to approve of my actions” became “I would like, and therefore I need, others to approve of my actions”), to maximize internal validity by making explicit the demand being measured, as well as including the positively phrased item, “I want to be, therefore I must be, in control of my emotions.” This was viewed favorably by the REBT practitioner, improving construct validity *a priori* (see Terjesen et al., 2009) and the remaining panels reported no other concerns. Ethical approval to collect data using the 86-item measure was received by the Ethics Committee of the University of the first author.

## Study 1: exploratory factor analysis

### Method

#### Participants

A final sample of 402 sports officials participated in the Exploratory Factor Analysis stage of the inventory development (*M*_age_ = 41.20, *SD* = 14.56). Of this sample, 349 were male, 50 were female, and three chose not to disclose their gender. As identification of officiating level is challenging due to varied classification across sports (see Webb et al., 2021), years’ experience of officiating was requested with the sample reporting with an average of 13.02 (*SD* = 10.27) years’ experience as a qualified sports official. The sample of participants comprised sports officials of 31 different nationalities, representing 5 continents and 11 sports (football, rugby union, rugby league, field hockey, netball, handball, cricket, GAA, baseball, badminton and futsal). While sample size for statistical validation analyses is disputed and frequently seen as arbitrary (see Brown, 2015), the final sample exceeded general guidelines of 200–300 observations (Boateng et al., 2018) and the recommendation of 5 participants per new item (5 × 65 = 325; DeVellis, 2012).

#### Procedure

Data collection took place using the online survey platform Qualtrics (Provo, UT). Links to surveys were distributed via social media (e.g., Twitter, LinkedIn) and personal emails to gatekeepers such as officiating development officers, Referee/Officiating Associations and national governing bodies. After confirming that they had read the participant information sheet, participants were asked to confirm their consent to participate in the research study. Participants completed an 86-item survey by indicating item response on a Likert-scale ranging from 1 (*strongly disagree*) to 5 (*strongly agree*), endorsed by Terjesen et al. (2009) to ascertain strength of belief.

#### Preliminary analysis

Data from an original sample of 513 participants was assessed for suitability prior to data analysis. One hundred and five responses were removed due to the absence of a significant amount of data points (e.g., participant dropout after commencing the study). Additionally, a further 6 responses were removed due to evidence of patterned responding, leaving 402 participants. From this sample, isolated cases of missing data were observed. Little’s Missing Completely at Random (MCAR) test indicated that items were not entirely missing at random, χ^2^ (2,705) = 2,268, *p* < 0.001, however removing incomplete cases was not considered prudent as (a) it would decrease the sample size and (b) the impact of data imputation (e.g., expectation maximation; where average scores are projected) is negligible when the percentage of missing data is lower than 5% (Schafer, 1999). As the largest percentage of missing data from any item was 1%^1^, and data imputation has been shown to improve efficiency of effect estimates compared to complete case analysis (Madley-Dowd et al., 2019), expectation maximization was used to impute missing data.

#### Data analysis

Reflecting the development and validation of the iPBI (see Turner et al., 2018a), the initial aim of analysis was to identify the factor structure of the measure and examine internal reliability, avoiding the assumption that the iPBI and new items conformed to the 4-factor structure. This was accomplished through EFA using SPSS Statistics 28 (Microsoft, Albuquerque, NM). Sampling adequacy and suitability of data for factor analysis was assessed using the Kaiser-Meyer-Olkin (KMO) measure and Bartlett’s test of sphericity. An EFA with oblique rotation (oblimin) was run on all 86 items. Items were retained if pattern matrix reported a primary factor correlation above 0.50 and secondary factor correlation below 0.30, as well as communality exceeding 0.40 (see Costello and Osborne, 2005; Field, 2009), with lowest scoring items removed in an iterative process. A maximum likelihood factor analysis was then conducted, with factors reporting eigenvalues greater than 1 deemed to meet the Kaiser’s criterion to determine the optimum factor solution for the data as per previous EFAs (e.g., Werner et al., 2014) and recommendations (e.g., Field, 2009). However, other sources (e.g., Velicer and Jackson, 1990) state that only retaining factors with eigenvalues greater than 1 lacks accuracy. Consequently, to determine the number of factors to extract, the scree test was used, identified as the optimum choice for researchers conducting an EFA (Costello and Osborne, 2005).

### Results

Sampling adequacy exceeded the desired value of greater than 0.70 (KMO = 0.940) and was categorized as “marvelous” (Lloret et al., 2017; Kaiser, 1974). Factor analyses was deemed suitable by Bartlett’s test of sphericity, χ^2^ (3,655) = 21570.00, *p* < 0.001. Fifty-two items were removed for failing to meet the criteria regarding correlation, factor loading and communality scores. Visual analysis of the scree plot, recommended as best practice to determine factor extraction and a strategy used in previous studies (Costello and Osborne, 2005; Turner et al., 2021; Watkins, 2018), suggested items loaded onto six-factors and therefore 6 factors were extracted. Results of the factor analysis for the remaining 22 items can be seen in Table 1.

**Table 1:** Exploratory factor analysis of the 22 retained items.

Items	Communalities			Factor				Eigenvalue	% variance explained	Dimension
		**1**	**2**	**3**	**4**	**5**	**6**			
I83DEP	0.56	**0.78**								Self-depreciation
I70DEP	0.60	**0.76**					−0.19			
I48DEP	0.56	**0.75**								
I22DEP	0.52	**0.71**		0.14	−0.17					
I14DEP	0.58	**0.70**		0.17			0.16	7.219	32.812	
I38DEP	0.59	**0.68**			0.15	0.15				
I53DEP	0.53	**0.67**				0.11	−0.17			
I58DEP	0.43	**0.62**								
I17DEP	0.47	**0.62**								
I9DEP	0.41	**0.60**			0.15	−0.10				
I79DEM	0.79		**0.92**							Peer rejection demands
I75DEM	0.56		**0.54**		0.19	0.11		2.499	11.359	
I78AWF	0.39		**0.51**			0.17				
I20DEM	0.53			**0.74**						Emotional control demands
I64DEM	0.57			**0.72**				1.952	8.871	
I33LFT	0.40			**0.55**	0.13		0.13			
I15DEM	0.65				**0.75**		−0.27			Approval
I5DEM	0.56				**0.70**			1.204	5.473	
I3AWF	0.54				**0.60**	0.10	0.29			
I32LFT	0.51	0.11			**0.57**					
I28AWF	0.62	0.23	0.43	0.32	0.25	**0.73**				Misc.
I26LFT	0.44		0.18			**0.54**		0.868	3.946	

Extraction method; maximum likelihood; Rotation method; oblimin with Kaiser normalization. Loadings larger than 0.60 are in bold. DEP, Depreciation; DEM, Demands; AWF, Awfulizing; LFT, Low frustration tolerance; Misc., Miscellaneous.

When items that loaded onto each factor were analyzed, clear dimensions were identified (Table 1). Four dimensions could be clearly identified as self-depreciation (e.g., “If I fail in things that matter to me, it means I am a failure”), peer rejection demands (e.g., “I do not want to be, therefore I must not be, dismissed by my peers”), emotional control demands (e.g., “I want to be, therefore I must be, in control of my emotions”) and approval (e.g., “I would like, and therefore I need, others to approve of my actions”). The fifth factor consisted of two items (“I do not want to, so I absolutely should not, fail in things that matter to me” and “It’s terrible if the members of my match-day team do not respect me”) that did not appear to be related and was labeled as “miscellaneous.” Although six factors were extracted, factor loadings indicated five clear factors (ranging from 0.60 to 0.78, 0.51 to 0.92, 0.55 to 0.74, 0.57 to 0.75, and 0.54 to 0.73 respectively).

### Discussion

The purpose of the EFA was to identify factors found within the measure. Six factors were extracted based on the scree plot being identified as the most advisable indicator (Costello and Osborne, 2005; Field, 2009), however the sixth factor was not the primary factor for any item. Therefore, the EFA suggested a five-factor model should be the initial model tested at the confirmatory factor analysis (CFA) stage. Although five factors were identified, it was unclear as to what the fifth factor was measuring based on visual analysis of its items (“I do not want to, so I absolutely should not, fail in things that matter to me” and “It’s terrible if the members of my match-day team do not respect me”) and was therefore categorized as “miscellaneous.” Following a “global assessment” (e.g., checking primary loadings and wording of items within each factor; see Costello and Osborne, 2005), the other four factors were clear and, based on the belief the items within the factors were measuring, categorized as: self-depreciation, peer rejection demands, emotional control demands, and approval. With the exception of self-depreciation, these factors differed from those identified in the iPBI. Although this was not hypothesized, identifying contextually relevant factors reflects the benefit of developing population-specific measures. Furthermore, although “approval” contained one item assessing low frustration tolerance and one item assessing awfulizing, their subordinate position to demands in the structure of irrational beliefs (see David et al., 2005; Dryden and Branch, 2008), suggesting that approval, the first or Ellis’ original 12 irrational beliefs (Ellis and Dryden, 1997), is of particular, contextual importance for sports officials.

Although the sample included participants for whom English was not their first language, leading to potential discrepancy in understanding measured constructs (see Chotpitayasunondh and Turner, 2019), an advantage of such diversity is the potential for generalization regarding the validity and reliability of the measure cross-culturally (Costello and Osborne, 2005).

## Study 2: confirmatory factor analysis and criterion validity

### Method

#### Participants

To avoid cohort effects a new sample was used to test model fit of the measure. This sample consisted of 154 participants (*M*_age_ = 39.92 years, *SD* = 16.26), 140 of whom were male, 12 were female, and two participants chose not to disclose their gender. Average years’ experience as a qualified sports official within the sample was 14.61 years (*SD =* 11.96), with participants officiating for an average of 2.95 h per week (*SD* = 1.14). Participants came from 12 different countries across four continents, and included officials from nine different sports (football, rugby union, rugby league, field hockey, basketball, baseball, cricket, Australian rules football and lacrosse). No missing data was reported in the sample, therefore the sample size exceeded the recommendation of 150 participants for CFA (Muthén and Muthén, 2002).

#### Procedure

As with the EFA stage, data was collected using the online survey platform Qualtrics (Provo, UT). Again, links to surveys were distributed via social media and personal emails to gatekeepers. Participants were instructed to only complete the survey had they not taken part in data collection for the EFA stage of inventory development and were asked to check an option to confirm that they had not completed the original survey. Once informed consent was given, participants completed the surveys by indicating item response on a 3, 4, or 5-point scale depending on the measure. This survey consisted of the 22 retained items of the new measure following the EFA, as well as items included from measures that assessed theoretical correlates. Additional measures were added at this stage to maximize the sample used to assess criterion validity.

#### Measures

##### Irrational beliefs scale for sports officials

The irrational beliefs scale for sports officials (IBSSO) consisted of 22 statements, with participants required to identify the extent to which they agreed with each using a 5-point scale (1 = *strongly disagree* to 5 = *strongly agree*). A score for each factor was determined, along with a composite score for the measure, with higher scores indicating stronger irrational demands and self-depreciation.

##### Positive mental health scale

The positive mental health scale (PMH-scale) (Lukat et al., 2016) consists of 9 items measuring proximal (e.g., emotional) and distal (e.g., social support) factors that contribute to positive mental health, using a four-point scale (1 = *not true* to 4 = *true*). The PMH-Scale reports high internal consistency (Cronbach’s α = 0.93), good test–retest reliability (*r* = 0.77), convergent and discriminant validity ranging from 0.26 to 0.81, and is easy to interpret (Lukat et al., 2016). These qualities, along with its assessment of adaptive emotional and behavioral responses, justified the choice to use this as a measure to compare with the IBSSO.

##### Patient health questionnaire

The patient health questionnaire (PHQ-9) (Kroenke et al., 2001) is a commonly used and validated measure to assess depression in primary care (Cameron et al., 2008). Comprised of 9 items, participants indicate the severity of depression symptoms using a four-point scale (1 = *not at all* to 4 = *every day*). The PHQ-9 reports excellent internal reliability (Cronbach’s α = 0.89) and excellent test–retest reliability (*r* = 0.84; Kroenke et al., 2001). The assessment of depression severity was chosen to evaluate the criterion validity of the self-depreciation items within the IBSSO.

##### Perth emotional reactivity scale-short form

The PERS-S (Preece et al., 2018) is a shortened version of the Perth Emotional Reactivity Scale (PERS; Becerra et al., 2017), a measure that distinguishes between positive and negative trait emotional reactivity and reports good to excellent concurrent validity (*r*’s from 0.80 to 0.98) and internal reliability (Cronbach’s α ranging from 0.79 to 0.94 for all factors; Becerra et al., 2017). The shortened version (consisting of 18-items measured using a 5-point scale) was chosen to assist the evaluation of criterion validity to maximize likelihood of survey completion, coupled with its assessment of ease and intensity of emotional activation.

##### Automatic thoughts questionnaire

The automatic thoughts questionnaire (ATQ-15) (Netemeyer et al., 2002) is a 15-item questionnaire that requires participants to assess the degree of believability across a range of cognitions using a 5-point scale (1 = *not at all* to 5 = *all the time*). The original 30-item inventory (Hollon and Kendall, 1980) reported good to excellent test–retest reliability and good internal stability (Cronbach’s α = 0.97) with a non-clinical sample. It is also frequently used as a measure of therapeutic progress (see Zettle et al., 2011). The 15-item survey was chosen to maximize response rate and the ATQ-15 also reports a negative correlation with self-esteem (*r* = 0.63), and a positive correlation with social anxiety (*r* = 0.56) and obsessive thoughts (*r* = 0.70; Netemeyer et al., 2002). Its correlation with obsessive thoughts, combined with its assessment of rigid (obsessive) cognitions, informed its selection to compare with the IBSSO.

##### Affective reactivity index

To assess criterion validity of low frustration tolerance within the IBSSO, the affective reactivity index (ARI) (Stringaris et al., 2012) was selected. Comprised of six items to assess irritability using a 3-point scale, the ARI reports high self-reporting internal reliability (Cronbach’s α = 0.90) and has been validated with UK and US samples, reflecting the international sampling regarding the development of the IBSSO.

##### The social phobia inventory

The social phobia inventory (SPIN) (Connor et al., 2000) is a 17-item self-reported survey that asks participants to indicate the extent to which they have been disturbed by symptoms of social anxiety during the previous 7 days on a 5-point scale (1 = *not at all* to 5 = *extremely*). The measure assesses a range of factors associated with social phobias, with fear of talking to strangers, criticism, authority figures, public speaking, physiological distress and negative social evaluation identified as factors (Connor et al., 2000; Campbell-Sills et al., 2015). The SPIN reports good concurrent validity (*r* = 0.57) with similar measures, such as the Liebowitz Social Anxiety Scale (LSAS; Liebowitz, 1987) and the Brief Social Phobia Scale (BSPS; Davidson et al., 1997), and excellent internal consistency (Cronbach’s α = 0.95; Connor et al., 2000). The significance of the factors identified within the survey justified its selection in comparison with the IBSSO.

#### Data analysis

A confirmatory factor analysis (CFA) was utilized due to five key advantages identified by Brown (2015). First, while the new measure was grounded in theory, there is a requirement for empirical evidence to accept, modify, or reject a measure (see Credé and Harms, 2019). Confirmation of the internal reliability measure of the measure can also decrease the likelihood of Type I and Type II errors, hence the need for statistical analysis (see Credé et al., 2012). Second, as REBT is multifactorial, analysis of both compound and subscale scores is desirable and supported by CFA. Third, CFA estimates scale dimensions allowing for tests of scale reliability, a traditional weakness in reporting practices of new measures (cf. Jackson et al., 2009; Terjesen et al., 2009). Fourth, CFA can be used to produce convergent and discriminant validity, required as irrational beliefs are a multidimensional construct. Finally, CFA allows for the generalization of groups, allowing the measure to identify prevalence of irrational beliefs on specific populations which has been identified as limited and in need of development (Turner, 2014; Turner et al., 2018a).

To overcome difficulties in selecting a goodness of fit index (e.g., contextual influence such as sample size on index choice, cut-off values etc.; see Hu and Bentler, 1999), multiple fit indices were identified for use: absolute fit, parsimony correction and comparative fit. Hence, goodness of fit was analyzed using the χ^2^ statistic, standardized root mean square residual (SRMR), root mean square error of approximation (RMSEA) and the comparative fit index (CFI). For transparency, values to establish reasonably good fit between the three, four and five-factor structures of the new measure and obtained data was identified as close to or below 0.08 for the SRMR, close to or below 0.06 for the RMSEA and close to or greater than 0.95 for the CFI. These values are consistent with guidelines outlined by Brown (2015) and those used to validate the original iPBI (Turner et al., 2018a). Internal reliability coefficients (Cronbach’s α) for each factor were also calculated, with coefficients exceeding 0.70 indicating good test score reliability (Nunnally and Bernstein, 1994). Data was analyzed using SPSS AMOS v28 (Microsoft, Albuquerque, NM), with three, four and five-factor models being tested for best fit. To develop model fit, an iterative process whereby the item that reported the lowest standardized factor loading was removed until goodness of fit was acceptable. Analysis began with testing the five-factor model, with an iterative process using the identified fit indices driving further analyses. A five-factor, four-factor, and three-factor model were subsequently tested. To analyze criterion validity, subscales of the new measure were correlated with the additional measures, as well as with participant age, experience and hours per week spent officiating. Positive correlations were expected between the IBSSO and the PERS-S, PMQ-9, ATQ-15, ARI and SPIN measures. Negative correlations were expected between the IBSSO and age, experience and the PMH-Scale.

### Results

#### Construct validity

Initial analysis of the 22-item five-factor model produced an insufficient fit according to the criteria, χ^2^(199) = 312.68, *p* = 0.001, SRMR = 0.08, CFI = 0.86, RMSEA = 0.07. The iterative removal of 5 items to improve model fit resulted in the removal of the fifth factor entirely, hence final model fit could not be identified, and the model was rejected.

Initial analysis of the four-factor model produced an unacceptable fit, χ^2^(203) = 341.27, *p* = < 0.001, SRMR = 0.08, CFI = 0.84, RMSEA = 0.08. An iterative process of removing the lowest loading items, however, led to model fit. In addition to the two items that loaded onto the fifth factor, four items (“It’s awful not to be treated fairly by my peers,” “I cannot stand not being in control of my emotions,” “If I do not act responsibly, it shows I am worthless” and “If I fail in things that matter to me, it means I am a failure”) were removed after reporting factor loadings of 0.409, 0.502, 0.495 and 0.564, respectively. This 16-item model produced an acceptable model fit, χ^2^(99) = 138.23, *p* = 0.006, SRMR = 0.06, CFI = 0.95, RMSEA = 0.05, standardized factor loadings were between 0.51 and 1.06 and error variances were between 0.26 and 1.13. Internal consistency (alpha reliability) coefficients were between 0.54 and 0.88, meaning three of the four factors were between acceptable and good, and one (Peer Rejection Demands) was below the acceptable range (0.70; Field, 2009).

Initial analysis of the three-factor model produced an unacceptable fit, χ^2^(101) = 154.18, *p* = 0.001, SRMR = 0.08, CFI = 0.90, RMSEA = 0.07. Iteratively, three low factor loading items were removed (“It’s awful not to be treated fairly by my peers,” 0.395; “If I do not act responsibly it shows I am worthless,” 0.497; and “I cannot stand not being in control of my emotions,” 0.527) and a subsequent CFA containing the remaining 13 items produced an acceptable fit to the three-factor structure suggested from the EFA, χ^2^(52) = 56.72, *p* = 0.303, SRMR = 0.05, CFI = 0.99, RMSEA = 0.02. For the 13-item measure, standardized factor loadings were between 0.58 and 1.09 and error variances were between 0.33 and 1.18. Internal consistency (alpha reliability) coefficients were between 0.59 and 0.85, meaning three of the four factors reported acceptable to good consistency, and one (Peer Rejection Demands) was below the acceptable range (0.70; Field, 20,019).

The identification of an item with a factor loading greater than 1 in both models (item 64: “I want to be, therefore I must be, in control of my emotions”) represents a likely Heywood case (see Farooq, 2022). The preferred matrix for analysis (Watkins, 2018), the pattern matrix, did not indicate this factor as poorly defined (see Table 1) and therefore equality constraints were imposed on both items in the factor, a common action buttressed by previous literature (Jung and Takane, 2008; Mustaffa et al., 2018). Standardized factor loadings then ranged from 0.54 to 0.81 and 0.58 to 0.80, with error variances between 0.33 to 0.61, and 0.33 to 0.65, for the 4-factor and 3-factor models, respectively (Tables 2, 3).

**Table 2:** Standardized solution and fit statistics for the four-factor 16-item model of the IBSSO (statistics in parentheses indicate results following application of equality constraints).

		Standardized factor loadings			
Items (original item number and original core belief)	Error variances	Self-depreciation	Peer rejection demands	Emotional control demands	Approval
If my competencies did not continually develop and improve it would show what a failure I am (I83:DEP)	0.33	0.58			
If I am not given opportunities, then it shows that I am not a worthwhile person (I70:DEP)	0.50	0.71			
If I am snubbed by people that matter to me, it means I am a worthless person (I48:DEP)	0.51	0.72			
If others think I do not make a valuable contribution, then I am no good (I38:DEP)	0.43	0.66			
If I am dismissed by my peers, then that makes me a loser (I53:DEP)	0.64	0.80			
If I am not treated with consideration, it shows I am unlikeable (I58:DEP)	0.46	0.68			
If people think I cannot control others it shows I am worthless (I17:DEP)	0.40	0.64			
If others are not considerate toward me, then it means I am worthless (I9:DEP)	0.51	0.71			
I do not want to be, therefore I must not be, dismissed by my peers (I79:DEM)	0.35		0.59		
I do not want to be, so absolutely should not be, snubbed by people that matter to me (I75:DEM)	0.40		0.63		
I would like to, and therefore I must, avoid losing control of my emotions (I20:DEM)	0.52			0.72	
I want to be, therefore I must be, in control of my emotions (I64:DEM)	0.57			0.76	
I want to be, therefore I need to be, approved of by others (I15:DEM)	0.65				0.81
I would like, and therefore I need, others to approve of my actions (I5:DEM)	0.61				0.78
It’s terrible if others think I am not good at officiating (I3:AWF)	0.29				0.54
I could not stand it if others disapproved of me (I32:LFT)	0.36				0.60

Factor	Mean ± SD	α (reliability coefficient)	Inter-factor correlations		
Self-depreciation (DEP)	18.63 ± 5.84	0.88			
Peer rejection demands (PRD)	6.35 ± 1.60	0.54	0.54**		
Emotional control demands (ECD)	7.94 ± 1.38	0.70	0.19**	0.39**	
Approval (A)	11.28 ± 3.33	0.77	0.68**	0.65**	0.06**

χ^2^(99) = 138.23, *p* = 0.006, SRMR = 0.06, CFI = 0.95, RMSEA = 0.05. DEM, Demands; DEP, Self-depreciation; LFT, Low frustration tolerance; AWF, Awfulizing.

**Table 3:** Standardized solution and fit statistics for the three-factor 13-item model of the IBSSO (statistics in parentheses indicate results following application of equality constraints).

		Standardized factor loadings		
Items (original item number and original core belief)	Error variances	Self-depreciation	Peer rejection demands	Emotional control demands
If my competencies did not continually develop and improve it would show what a failure I am (I83:DEP)	0.34	0.58		
If I am not given opportunities, then it shows that I am not a worthwhile person (I70:DEP)	0.52	0.72		
If I am snubbed by people that matter to me, it means I am a worthless person (I48:DEP)	0.53	0.73		
If I fail in things that matter to me, it means I am a failure (I14:DEP)	0.32	0.57		
If others think I do not make a valuable contribution, then I am no good (I38:DEP)	0.41	0.64		
If I am dismissed by my peers, then that makes me a loser (I53:DEP)	0.65	0.80		
If I am not treated with consideration, it shows I am unlikeable (I58:DEP)	0.48	0.69		
If people think I cannot control others it shows I am worthless (I17:DEP)	0.40	0.63		
If others are not considerate toward me, then it means I am worthless (I9:DEP)	0.49	0.70		
I do not want to be, therefore I must not be, dismissed by my peers (I79:DEM)	0.33		0.58	
I do not want to be, so absolutely should not be, snubbed by people that matter to me (I75:DEM)	0.42		0.64	
I would like to, and therefore I must, avoid losing control of my emotions (I20:DEM)	0.52			0.72
I want to be, therefore I must be, in control of my emotions (I64:DEM)	0.57			0.76

Factor	Mean ± SD	α (reliability coefficient)	Inter-factor correlations	
Self-depreciation (DEP)	21.10 ± 6.01	0.85		
Peer rejection demands (PRD)	6.35 ± 1.60	0.59	0.53	
Emotional control demands (ECD)	7.94 ± 1.38	0.71	0.16	0.24

χ^2^(52) = 56.72, *p* = 0.303, SRMR = 0.05, CFI = 0.99, RMSEA = 0.02. DEM = Demands; DEP = Self-depreciation.

#### Concurrent validity

Correlations between the 16-item IBSSO, 13-item IBSSO, and other measures were assessed to test concurrent validity (Tables 4, 5 respectively). Low to medium correlations were mostly reported, with the largest correlations for the self-depreciation factor found between the SPIN (*r* = 0.44 for both models) and the ATQ-15 (*r* = 0.42 and *r* = 0.43 for the 4 and 3-factor models respectively). The composite score of the 4-factor model was significantly and positively correlated with all measures except the ARI, and negatively correlated with the PMH-Scale, supporting concurrent and convergent validity of this model in evaluating mental well-being in officiating populations. Similar correlations were found in the 3-factor model, although the relationship between the compound score of the measure and the PERS-S was not significant. As hypothesized, age was negatively correlated with irrational beliefs in sports officials (*r* = −0.18 and *r = −0*.25 in the 4 and 3-factor models respectively), although age was a more accurate predictor of levels of self-depreciation than other factors (*r* = −0.27 in both models). Experience was also negatively correlated with irrational beliefs in sports officials (*r =* −0.04 and *r = 0*.06 in the 4 and 3-factor models respectively), however this variable was not as significant a predictor of irrational beliefs as age.

**Table 4:** Descriptive data of the Positive Mental Health Scale (PMH-Scale), Patient Health Questionnaire (PHQ-9), Perth Emotional Reactivity Scale-Short Form (PERS-S), Automatic Thoughts Questionnaire (ATQ-15), Affective Reactivity Index (ARI), Social Phobia Inventory (SPIN), demographic related variables, and correlations with the 16-item 4-factor model dimensions.

Measures	Mean ± *SD*	α (reliability coefficient)	Self-depreciation	Peer rejection demands	Emotional control demands	Approval	IBSSO composite score
PMH-scale	31.11 ± 4.12	0.87	−0.36**	−0.55	0.04	−0.20*	−0.29**
PHQ-9	13.41 ± 4.45	0.84	0.30**	0.15	0.04	0.07	0.22*
PERS-S	58.70 ± 8.67	0.77	0.23*	0.12	−0.08	0.23*	0.20*
ATQ-15	21.82 ± 7.12	0.90	0.42**	0.12	0.08	0.21*	0.35**
ARI	7.65 ± 2.29	0.86	−0.04	0.02	−0.18	0.01	−0.08
SPIN	28.66 ± 10.77	0.92	0.44**	0.06	0.11	0.27**	0.40**
Demographics							
Age	39.54 ± 16.55	**-**	−0.27**	−0.05	−0.02	−0.01	−0.18
Experience	12.92 ± 10.96	**-**	−0.07	−0.01	0.03	0.04	−0.04
Hours	2.95 ± 1.14	**-**	−0.05	−0.11	0.00	−0.03	−0.04

*n* = 111, *p < 0.05, **p < 0.01. 16-item 4-factor model composite score: mean = 41.95, SD = 9.56, α = 0.87. Contents of scales: PMH-Scale = antecedents to positive mental health; PHQ-9 = Depression; PERS-S = Positive and Negative trait emotional reactivity; ATQ-15 = Self-esteem and obsessive thoughts; ARI = irritability; SPIN = social anxiety. Hours = hours per week officiating.

**Table 5:** Descriptive data of the Positive Mental Health Scale (PMH-Scale), Patient Health Questionnaire (PHQ-9), Perth Emotional Reactivity Scale – Short Form (PERS-S), Automatic Thoughts Questionnaire (ATQ-15), Affective Reactivity Index (ARI), Social Phobia Inventory (SPIN), demographic related variables, and correlations with the 13-item 3-factor model dimensions.

Measures	Mean ± *SD*	α (reliability coefficient)	Self-depreciation	Peer rejection demands	Emotional control demands	IBSSO composite score
PMH-scale	31.11 ± 4.12	0.87	−0.38**	−0.55	0.04	−0.32**
PHQ-9	13.41 ± 4.45	0.84	0.30**	0.15	0.04	0.30**
PERS-S	58.70 ± 8.67	0.77	0.21*	0.12	−0.08	0.18
ATQ-15	21.82 ± 7.12	0.90	0.43**	0.12	0.08	0.38**
ARI	7.65 ± 2.29	0.86	−0.04	0.02	−0.18	−0.08
SPIN	28.66 ± 10.77	0.92	0.44**	0.06	0.11	0.40**
Demographics						
Age	39.54 ± 16.55	**–**	−0.27**	−0.05	−0.02	−0.25**
Experience	12.92 ± 10.96	**–**	−0.07	−0.01	0.03	−0.06
Hours	2.95 ± 1.14	**–**	−0.05	−0.11	0.00	−0.04

*n* = 111, *p < 0.05, **p < 0.01. 13-item 3-factor model composite score: mean = 37.56, SD = 7.56, α = 0.85. Contents of scales: PMH-Scale = antecedents to positive mental health; PHQ-9 = Depression; PERS-S = Positive & Negative trait emotional reactivity; ATQ-15 = Self-esteem & obsessive thoughts; ARI = irritability; SPIN = social anxiety. Hours = hours per week officiating.

### Discussion

The CFA produced acceptable model fit for both the 4-factor and 3-factor models of the IBSSO, and the correlation between both models and measures that assess similar constructs support both the concurrent and convergent validity of both models. For example, considering the similar constructs evaluated in the self-depreciation factor extant in both models and the SPIN and ATQ-15 (e.g., depression, fear of evaluation and rigid, obsessive thoughts), a significant and positive correlation is not surprising. A small but negative correlation was found between ARI and the IBSSO. While this was not expected *a priori,* the 4 and 3-factor models only containing one or no LFT items, respectively, supports the convergent validity of the IBSSO (e.g., there are no factors that measure LFT). Additionally, it was hypothesized age would be positively association with irrational beliefs (Ndika et al., 2012; Turner et al., 2019), a result found in both models supporting the concurrent validity of the measure.

Although the 3-factor model reported better fit, along with a larger and significant negative correlation with age, the level of significance (*p* = 0.303) alongside the elimination of the approval factor that consists of an awfulizing and low frustration tolerance item, means caution should be shown when interpreting the results. While models should be statistically sound, factor analysis alone is not sufficient to justify the removal of an item or factor that has theoretical importance (Flora and Flake, 2017). For example, while Bernard (1988) found a 7-factor fit for the original Attitude & Beliefs Scale (and thus was statistically supported), this was seen as having poor “theoretical fit,” justifying further investigation and the eventual development of the Attitudes and Beliefs Scale-2 (DiGiuseppe et al., 2020). As approval is one of the Ellis’ (1962) original irrational beliefs, and low frustration tolerance and awfulizing recognized secondary irrational beliefs (Dryden and Branch, 2008), the 4-factor model was accepted. This model reported acceptable fit and improved level of significance (*p* = 0.006). Additionally, the model preserves more items which is consistent with the recommended policy of conservatism in construct development (DeVellis, 2012) and theoretical alignment (Flora and Flake, 2017).

A limitation of both models is that two factors contain only two items, with a minimum of three items recommended for strong and stable factors (Costello and Osborne, 2005), potentially indicated by the reliability score of the Peer Rejection Demands (α = 0.54 and 0.59 for the 16- and 13-item models respectively) being below the 0.70 threshold recommended by Terjesen et al. (2009). However, Yang and Green (2011) state it is unlikely items contribute equally to a factor, an assumption of Cronbach’s alpha. Hence, Cronbach’s alpha should be treated as a lower-bound estimate of internal consistency, and true reliability could be up to 20% greater (Green and Yang, 2009; McNeish, 2017). Furthermore, underestimation of reliability is strongest when factors contain small number of items, therefore it is likely that true reliability is greater than what is reported (Graham, 2006).

While one item reported a factor loading and error variance of above 1, this was not judged to be a result of a poorly defined factor, as both items within the factor assess the primary irrational belief of demands with the subscale of emotional control. Theoretically, this is important as contemporary research identifies emotional control as a valuable attribute for sports officials (Carrington et al., 2022; McEwan et al., 2020). Hence, the presence of this Heywood case may be attributable to sample size as, although the sample meets requirements identified by Muthén and Muthén (2002), it is smaller than the minimum of 1,000 recommended by Boateng et al. (2018). Ultimately, the 4-factor model was considered the most suitable model of the IBSSO from a statistical and theoretical perspective.

## Study 3: convergent validity analysis

To develop the validity of the IBSSO, the aim of Study 3 was to test convergent validity of both acceptable models of the IBSSO against a similar measure.

### Method

#### Participants

A new sample was used to assess the convergent validity of the 16-item 4-factor model and the 13-item 3-factor model of the IBSSO. The sample consisted of 94 participants (*M*_age_ = 36.74 years, *SD* = 15.03), 83 of whom were male, 10 were female, and one participant chose not to disclose their gender. Average years’ experience as a qualified sports official was 13.89 years (*SD =* 10.88) across eight sports (football, rugby union, rugby league, field hockey, basketball, ice hockey, cricket, and lacrosse). To determine sample size G*Power (version 3.1.9.7; Heinrich-Heine-Universität Düsseldorf, Germany), recommended software to determine sample size to conduct power analysis (Kang, 2021), identified a minimum sample of 85 was required for a medium effect size (0.30), alongside power and α (*p* value) set at 0.80 and 0.05, respectively. Thus, the sample used for this study exceeds this recommendation.

#### Procedure

As with Study 1 and Study 2, data was collected using the online survey platform Qualtrics (Provo, UT), with links to surveys distributed via social media and personal emails to gatekeepers. Once informed consent was given by confirming the information sheet and consent form had been read and agreed to, participants completed the surveys by indicating item response on a 5-point Likert scale. The survey consisted of the 22-retained items of IBSSO, as well as items from the iPBI (Turner et al., 2018a) to assess convergent validity.

The iPBI is a 28-item measure that assesses the four core irrational beliefs: demandingness, awfulizing, low-frustration tolerance and depreciation (Dryden, 2014). Participants are asked to report the extent they agree or disagree with a statement using a 5-point Likert scale (1 = *strongly disagree* to 5 = *strongly agree*). The iPBI was chosen because of its good construct validity, χ^2^ (344) = 1433.98, *p* < 0.001, CFI = 0.93, NNFI = 0.92, SRMR = 0.06, RMSEA = 0.07, and internal consistency across factors (between 0.90 and 0.96; Turner et al., 2018a). Additionally, the iPBI reports good concurrent validity with measures of similar constructs. For example, there is a high correlation (*r = 0*.86, *p* < 0.001) between the depreciation factor of the iPBI and self-downing measured by the SGABS (Lindner et al., 1999), and predictive validity through small to medium correlations with trait subscales of the State–Trait Personality Inventory (STPI; Spielberger, 1979; Turner et al., 2018a). Furthermore, the iPBI evaluates irrational beliefs in a performance context, justifying its use to confirm the convergent validity of the IBSSO.

Upon completion of data collection, scores from the IBSSO and the iPBI were checked for incomplete data, patterned responses, and outliers. There were no incomplete cases nor any patterned responses. A Shapiro Wilks test was conducted with *z* scores to identify outliers. One score of above 3.29, found within the depreciation subscale of the iPBI, was identified and winsorized (Field, 2009). Data was correlated using SPSS Statistics 30 (Microsoft, Albuquerque, NM). High correlations were expected between the IBSSO and iPBI, along with high correlations between relevant subscales (e.g., depreciation factors of both measures).

### Results

As expected, high correlations were reported between the 16-item 4-factor model and the iPBI (*r = 0*.84, *p* < 0.01), and the 13-item 3-factor model and the iPBI (*r* = 0.81, *p* < 0.01; Tables 6, 7 respectively). The largest correlations across subscales was found between the depreciation factor of the iPBI and the self-depreciation factor of the IBSSO (*r = 0*.79, *p* < 0.01). Given the similar conceptualization of these factors, it is not surprising a high correlation was found. Demand-based factors of the IBSSO (peer rejection demands and emotional control demands) and the approval factor reported medium to high correlations with the demandingness factor of the iPBI.

**Table 6:** Descriptive data of the Irrational Performance Beliefs Inventory (iPBI), and its individual factors of Demandingness (DEM), Low-Frustration Tolerance (LFT), Awfulizing (AWF), Self-Depreciation (DEP), demographic related variables, and correlations with the 16-item 4-factor model dimensions.

iPBI and demographic variables	Mean ± *SD*	α (reliability coefficient)	Self-depreciation	Peer rejection demands	Emotional control demands	Approval	IBSSO composite score
iPBI	86.57 ± 18.70	0.95	0.74**	0.58**	0.38**	0.73**	0.84**
DEM	24.11 ± 4.54	0.78	0.50**	0.61**	0.31**	0.58**	0.65**
LFT	25.51 ± 5.73	0.89	0.55**	0.50**	0.59**	0.49**	0.67**
AWF	21.77 ± 5.55	0.86	0.65**	0.51**	0.25*	0.71**	0.75**
DEP	15.19 ± 5.99	0.92	0.79**	0.38**	0.17	0.71**	0.79**
Demographics							
Age	36.74 ± 15.03	**–**	−0.33**	−0.14	−0.36**	−0.15	−0.33**
Experience	13.89 ± 10.88	**–**	−0.13	0.02	−0.08	−0.05	−0.10

*n* = 94, *p < 0.05, **p < 0.01. 16-item 4-factor model composite score: mean = 43.12, SD = 10.39, α = 0.89.

**Table 7:** Descriptive data of the Irrational Performance Beliefs Inventory (iPBI), and its individual factors of Demandingness (DEM), Low-Frustration Tolerance (LFT), Awfulizing (AWF), Self-Depreciation (DEP), demographic related variables, and correlations with the 13-item 3-factor model dimensions.

iPBI and demographic variables	Mean ± *SD*	α (reliability coefficient)	Self-depreciation	Peer rejection demands	Emotional control demands	IBSSO composite score
iPBI	86.57 ± 18.70	0.95	0.74**	0.58**	0.38**	0.81**
DEM	24.11 ± 4.54	0.78	0.50**	0.61**	0.31**	0.62**
LFT	25.51 ± 5.73	0.89	0.55**	0.50**	0.59**	0.69**
AWF	21.77 ± 5.55	0.86	0.65**	0.51**	0.25*	0.70**
DEP	15.19 ± 5.99	0.92	0.79**	0.38**	0.17	0.76**
Demographics						
Age	36.74 ± 15.03	**–**	−0.33**	−0.14	−0.36**	−0.38**
Experience	13.89 ± 10.88	**–**	−0.13	0.02	−0.08	−0.12

*n* = 94, *p < 0.05, **p < 0.01. 13-item 3-factor model composite score: mean = 33.29, SD = 7.46, α = 0.83.

### Conclusion

The addition of this study and the high correlation found with a similar measure (the iPBI) supports the convergent validity of the IBSSO.

## Study 4: test–retest reliability

### Method

#### Participants

A new independent sample of 29 participants (*M*_age_ = 48.25 years, *SD* = 14.77), with an average of 14.57 years’ experience (*SD =* 12.44) as a sports official, completed the test–retest reliability study. Participants (25 male, 4 female) were from the United Kingdom and Ireland, the United States of America and Canada, and Australia, representing 6 sports (football, cricket, rugby union, baseball, field hockey and Australian Rules Football). Officials reported their level of practice as either recreational (*n* = 16), semi-professional (*n* = 8) or national/international (*n* = 5). While larger sample sizes have been recommended (see Kennedy, 2022), samples of between 25 and 30 are seen as sufficient by others, justified by personal experience and statistical theory, such as a medium effect size and sufficient power (Bonett and Wright, 2014; Clark-Carter, 2010; Cocchetti, 1999). Further, G*Power (version 3.1.9.7; Heinrich-Heine-Universität Düsseldorf, Germany) recommended a minimum sample size of 13 participants for a bivariate correlation analysis with 0.08 power and 0.05 α (*p* value).

**Table 8:** Repeated-measures ANCOVA, intra-class coefficients, means ± SD for sports official data across three data collection time points.

Officials’ Data	Means ± SD			Intra-class coefficients	
Variables	Time 1	Time 2 (7 days)	Time 3 (28 days)	ICC	95% CI
Officials’ data					
DEP	15.83 ± 6.94	16.45 ± 6.79	17.14 ± 6.11	0.85	0.75–0.92
PRD	5.76 ± 2.17	5.83 ± 2.02	6.17 ± 2.09	0.75	0.60–0.86
ECD	7.03 ± 2.08	7.31 ± 2.00	6.87 ± 2.15	0.71	0.55–0.84
APP	10.52 ± 4.17	10.41 ± 3.74	10.76 ± 4.16	0.83	0.71–0.91
COMP.	39.24 ± 13.66	40.10 ± 12.73	40.86 ± 11.95	0.89	0.82–0.94

Cronbach’s α ranges for each subscale: DEP = 0.86–0.96; PRD = 0.64–0.72; ECD = 0.70–0.94; APP = 0.83–0.95.

#### Procedure

The IBSSO, along with demographic information, was completed via Qualtrics (Provo, UT) over three-time points, with the second assessment after 7 days and the third assessment after another 21 days, replicating previous test–retest research (e.g., Turner et al., 2018b). This approach exceeds the two-time points recommended in literature (e.g., Law, 2004) and used previously to develop measures (e.g., SGABS, Lindner et al., 1999). Furthermore, a 28-day interval between the first and third assessments minimized the risk of respondents remembering previous answers, a concern in measuring retest reliability (Polit, 2014). After each survey participants were asked for their email address, with this information handled and stored according to General Data Protection Regulations (e.g., on the first author’s university server requiring a two-factor verification to log in), to correctly identify data and distribute the second and third surveys.

#### Data analysis

There was no missing data. A Shapiro Wilks test was conducted with *z* scores to identify outliers. Two scores of above 3.29, one from the first and one in the second-time point of the DEP subscale, were identified and winsorized (Field, 2009).

Data analyses were then conducted in two phases. First, a repeated-measures MANCOVA was performed to evaluate changes in each subscale of the IBSSO: self-depreciation (DEP); peer-rejection demands (PRD); emotional control demands (ECD); and approval (A), across the three timepoints, with no change in direction and non-significant results expected to demonstrate the reliability of the IBSSO. Consistent with literature identifying a negative relationship between age and irrational beliefs (see Ndika et al., 2012; Turner et al., 2019), age was included as a covariate. Second, intra-class coefficients (ICCs) were conducted, with scores above 0.7 in all subscales and compound scores applied to assess and establish consistency of irrational beliefs across three-time points.

### Results

The repeated-measures MANCOVA revealed no main effect of time, Wilk’s *λ* = 0.57, *F* (8, 20) = 1.89, *p* = 0.19, η^2^ = 0.43. Direction and non-significance of results was not affected by removing age as a covariate, Wilk’s λ = 0.62, *F* (8, 20) = 1.52, *p* = 0.21, η^2^ = 0.39. This indicates that there is no effect for time in any of the variables within the IBSSO.

For ICCs, all subscales and compound scores reported results above 0.7, demonstrating strong agreement and consistency across all three measurements points for all variables (Table 8). This indicates that the IBSSO is not influenced by time.

## General discussion

The aim of this study was to develop, validate and test the reliability of a specific measure of irrational beliefs in sports officials. An EFA supported analysis of a five, four and three-factor model. Consequently, CFA rejected the five-factor model but supported the four and three-factor models. Low to medium correlations between factors of the IBSSO and existing measures of related emotional dimensions supported criterion validity in the three and four-factor models. Specifically, self-depreciation and the composite score of the IBSSO was negatively correlated with mental well-being, and positively correlated with depression and related social phobias. Predictive validity was established through expected negative correlations with age, consistent with previous research (Ndika et al., 2012), and good test–retest reliability was demonstrated, with stable scores for subscales and compound score reported across three-time points.

The 13-item, three-factor model reported strong model-fit and contained items that measured demands and self-depreciation. While this was not expected, as the inclusion of items that assessed low frustration tolerance and awfulizing was anticipated due to their presence in extant measures and theoretical significance (Dryden and Branch, 2008; Turner et al., 2018a), this finding is less surprising when the nature of irrational beliefs is considered. While Ellis (1962) and others (e.g., Dryden, 1984) consider irrational beliefs to be evaluative cognitions, with demands being the core irrational belief leading to secondary irrational beliefs, a distinction has previously been made between appraising (labeled as “hot” cognitions) and knowing (labeled as “cold” cognitions; Abelson and Rosenberg, 1958). Cold cognitions are subsequently categorized into surface cognitions (descriptions, attributions and inferences) and deep cognitions refer to core beliefs, or schemata, that specify the value that any given event has on a range of appraisals (Anderson, 2000; Szentagotai et al., 2005). This view is endorsed by DiGiuseppe (1996), who posited that irrational beliefs are “evaluative schemas” that represent an individual’s concept of reality which, owing to the constructivist nature of this claim, strengthens the need for bespoke measures for specific populations. Moreover, demandingness and self-depreciation have been classified as evaluative schemas, while low frustration tolerance and awfulizing have been identified as appraisals (see David et al., 2005) and therefore measures that assess demandingness and self-depreciation may be more beneficial in revealing core beliefs. While Szentagotai et al. (2005) suggest DiGiuseppe’s (1996) distinction between schemata and appraisals should be treated with caution, as it is “based solely on logical analyses and clinical observations, without any empirical evidence to support these assertions” (p. 142), the same can be said of Ellis’ (1962) structuring of primary and secondary irrational beliefs (David et al., 2005). Additionally, DiGiuseppe et al. (1988) found that demandingness, awfulizing and low frustration tolerance loaded onto one factor during a CFA, with self-depreciation loading onto another, providing further support for the findings reported here.

The identification of self-depreciation loading onto one factor, with demandingness, awfulizing and low frustration tolerance loading onto another is also seen in the four-factor model. As seen in Table 2, items measuring self-depreciation load onto factor one (labeled depreciation), with items loading onto the remaining factors (peer rejection demands, emotional control demands and approval) assessing demandingness, awfulizing and low frustration tolerance. The three-factor model’s *p* value (0.054), alongside the good reliability coefficient of the fourth factor (approval) and the policy of conservatism recommended by DeVellis (2012), led to the rejection of the three-factor model in favor of the four-factor alternative.

The identification of the factors within this model is supported in previous literature regarding REBT and sports officials, self-depreciation was an expected factor due to its prominence in REBT literature (e.g., Ellis, 1962, 1988; Dryden and Branch, 2008). Contemporary support for the impact of self-depreciation on maladaptive outcomes can be found in recent investigation into perfectionism in sport. When exploring sport psychology consultants’ experiences with athletes who demonstrated perfectionist behaviors, Klockare et al. (2022) reported that such athletes hold the belief that they must be perfect or they are “nothing.” While this perspective is reported as an attitude, this is what Dryden (2016) has previously labeled irrational beliefs and, as Klockare et al. (2022) sought out perspectives from practicing sport psychology consultants, the terms “attitude” and “beliefs” likely hold lexiconic similarities. Additionally, such attitudes were identified as having undesirable effects on performance. For instance, one of the consultants discusses how an athlete holding this belief feels their time is wasted if they make a mistake in the first minute of competition as they can no longer be perfect. Such a belief would have a significant effect on the performance of sports officials, as another consultant states that a referee in possession of such a belief will fixate on an error, interfering with future performance (Klockare et al., 2022). Additionally, two rugby union officials reported a 31.45% reduction in demandingness, awfulizing and low frustration tolerance after an REBT intervention, while self-depreciation scores only reduced by 8.75% (Maxwell-Keys et al., 2022). Therefore, self-depreciation appears to be a robust irrational belief among sports officials. As elite performers report lower levels of self-depreciation than recreational performers and non-athletes (Turner et al., 2019), and REBT has shown promise in reducing self-depreciation in sporting populations (e.g., Cunningham and Turner, 2016), the measurement and reduction of this secondary irrational beliefs is particularly valuable for enhancing the performance within sporting populations.

Support for the identification of emotional regulation as a factor that officials may hold irrational demands about can be seen in research that acknowledges its importance to performance. For example, calmness (and the avoidance of stress) has been classified as an emotion that contributes to accurate decision-making in sports officials (Anshel and Weinberg, 1995; Castillo-Rodriguez et al., 2021; Neil et al., 2013), with lacrosse and rugby union officials adopting emotion-focussed coping strategies to maintain optimum performance (Friesen et al., 2017; Hill et al., 2016). A possible explanation for the importance of emotional regulation within this population is that it is seen as a source of efficacy specific to officiating (see Guillén and Feltz, 2011). The importance of positive resource appraisal (e.g., being able to control emotions) may contribute to more positive emotional and behavioral outcomes (see Dixon and Turner, 2018), and the promotion of challenge states that has been shown to reduce feelings of anxiety in sports officials (Noguiera et al., 2022). Therefore, the value of effective emotional regulation may promote irrational beliefs (e.g., “I want to, and therefore I must, be in control of my emotions”) in officials.

The final factors of peer rejection demands and approval are well established in officiating research. The construction of the Referees Stress Questionnaire (RSQ; Gomes et al., 2021) identified the receipt of negative evaluations from official observers and the need to be able to control the behavior of others as sources of stress for officials. The importance of significant others (e.g., official observers) was supported during the construction of IBSSO, and evaluation from assessors and colleagues (prior to and during performance) has been identified as an emotion-eliciting event for officials (Friesen et al., 2017). Additionally, the approval of others may also contribute to irrational beliefs held by sports officials. Neil et al. (2013) identified that less experienced officials may sacrifice decision-making accuracy to appease, or avoid criticism from, others. The impact of social pressures has also been found at higher levels of officiating, with social bias contributing to inaccuracy regarding time-added on and disciplinary sanctions (see Dohmen and Sauermann, 2016, for a review). The significant correlation between the SPIN and the IBSSO suggests that fear of negative social evaluation is a factor that influences the beliefs (and, by extension, the performance and well-being) of sports officials.

The development of the IBSSO provides researchers with a bespoke measure of irrational beliefs within officiating populations. However, it is important to highlight some limitations regarding its development. First, it does not meet *all* recommendations made by Terjesen et al. (2009) regarding inventory construction. For example, there are not an equal number of items for each factor and the assessment of rational beliefs is absent. The unequal number of items may be responsible for the low (α = 0.54) internal reliability of the peer rejection demands factor. However, as stated, this may be because Cronbach’s α assumes equal contribution of items (Yang and Green, 2011). Further, it may be prudent to assess factors with a low number of items differently to other factors, with Cronbach’s α between 0.45 and 0.60 previously reported as “acceptable” with limited items (Berger and Hänze, 2015). Additionally, the measure does not contain the same number of irrational beliefs, with more items for self-depreciation and demandingness than others. However, as stated by Terjesen et al. (2009), this is permissible should an irrational belief be of more clinical use than others, justifying their over-representation. When five REBT practitioners were asked which irrational beliefs interfere with performance the most, all five reported demandingness and self-depreciation as most significant, with these beliefs also impacting an individual’s ability to recover from major adversities (e.g., injuries; Turner et al., 2022). Hence, demandingness and self-depreciation are of greater utility from a clinical perspective than other irrational beliefs. Second, the IBSSO is based on the self-reporting of irrational beliefs, identified as a limitation of existing measures with other methods, such as implicit testing, recommended (David et al., 2005; Ellis, 1996; Jordana et al., 2020).

Looking forward, four recommendations are made to develop and use the IBSSO. First, to establish criterion validity, evaluation of sports officials’ performance using observers and objective criteria is recommended to overcome the reliance on self-reporting measures (Jordana et al., 2020). Second, to maintain a strong factor with less than two items, future research that utilizes the IBSSO should replicate these studies and recruit large samples (Costello and Osborne, 2005). In particular, the recommendation of 10 participants per new item (Boateng et al., 2018) is advised. This could be achieved by addressing the third recommendation, greater investigation of the IBSSO with female officials (who made up less than 12% of the total sample used across the four studies), particularly as females are more likely to experience negative events (e.g., sexism) when officiating (Tingle et al., 2014). Finally, while the use of scree plot is identified as the optimum method of factor extraction (Costello and Osborne, 2005; Watkins, 2018), justifying its application, additional EFA extracting different factors may assist in further development and validation of the IBSSO (see Fabrigar et al., 1999). While further validation studies of the IBSSO would be welcome, the method of development employed here minimized the impact of cohort effects (e.g., a new sample was recruited for CFA following EFA, as well as for Study 3 and Study 4) and recruited large samples that are essential for effective EFA (Costello and Osborne, 2005). Additionally, requirements for transparent reporting regarding item development and preliminary analysis (see Hughes et al., 2019; Jackson et al., 2009) and requirements for goodness of fit being met in the CFA (Brown, 2015) were fulfilled. Furthermore, the concurrent validity, convergent validity, and test–retest reliability of the IBSSO established in this paper means researchers are provided with a valid and reliable tool for assessing irrational beliefs in sports officials.

## Data Availability

The raw data supporting the conclusions of this article will be made available by the authors, without undue reservation.

## Footnotes

^1^Analysis of missing item data is available upon request.
